# SARS-CoV-2-induced Overexpression of miR-4485 Suppresses Osteogenic Differentiation and Impairs Fracture Healing

**DOI:** 10.7150/ijbs.56657

**Published:** 2021-03-25

**Authors:** Bobin Mi, Yuan Xiong, Chenming Zhang, Wu Zhou, Lang Chen, Faqi Cao, Fenghua Chen, Zhi Geng, Adriana C. Panayi, Yun Sun, Lin Wang, Guohui Liu

**Affiliations:** 1 Department of Orthopedics, Union Hospital, Tongji Medical College, Huazhong University of Science & Technology Wuhan, Hubei 430022, China.; 2 Department of Clinical Laboratory, Union Hospital, Tongji Medical College, Huazhong University of Science & Technology Wuhan, Hubei 430022, China.; 3 Division of Plastic Surgery, Brigham and Women's Hospital, Harvard Medical School, Boston 02115, USA.; 4 Department of Neurosurgery, Union Hospital, Tongji Medical College, Huazhong University of Science & Technology Wuhan, Hubei 430022, China.

**Keywords:** SARS-CoV-2, miR-4485, Fracture, Differentiation, Osteoblast

## Abstract

The angiotensin-converting enzyme 2 (ACE2) receptor has been identified as the cell entry point for SARS-CoV-2. Although ACE2 receptors are present in the bone marrow, the effects of SARS-CoV-2 on the biological activity of bone tissue have not yet been elucidated. In the present study we sought to investigate the impact of SARS-CoV-2 on osteoblastic activity in the context of fracture healing. MicroRNA-4485 (miR-4485), which we found to be upregulated in COVID-19 patients, negatively regulates osteogenic differentiation. We demonstrate this effect both *in vitro* and* in vivo*. Moreover, we identified the toll-like receptor 4 (TLR-4) as the potential target gene of miR-4485, and showed that reduction of TLR-4 induced by miR-4485 suppresses osteoblastic differentiation *in vitro*. Taken together, our findings highlight that up-regulation of miR-4485 is responsible for the suppression of osteogenic differentiation in COVID-19 patients, and TLR-4 is the potential target through which miR-4485 acts, providing a promising target for pro-fracture-healing and anti-osteoporosis therapy in COVID-19 patients.

## Introduction

The severe acute respiratory syndrome coronavirus 2 (SARS-CoV-2) is the novel human respiratory viral infection causing COVID-19 which has rapidly progressed into a life-threatening pandemic with significant morbidity and mortality 1. Around 5% of COVID-19 patients experience severe symptoms including acute respiratory distress syndrome (ARDS), septic shock, and organ dysfunction. Most patients, however, have mild symptoms 2. Although the underlying mechanisms of this impairment remain elusive, the angiotensin-converting enzyme 2 (ACE2) receptor has been identified as the necessary cell entry point for SARS-CoV-2 3. The proteins encoded by this gene belong to the ACE family of dipeptidase carboxylic dipeptidase and have considerable homology with human ACE 1 4. ACE2 has a strong affinity for Angiotensin II type 1 and Type 2 receptors and modulates inflammation, fluid balance, blood pressure, cell proliferation, fibrosis, and hypertrophy 5. Furthermore, specific expression of this gene in organs and cells has been shown to play a role in regulating cardiovascular and renal function as well as fertility 6. In addition, the gene encoding the protein is a functional receptor for the human coronavirus S glycoprotein of SARS and HCOV-NL63 7.

For some organs, ACE2 receptor expression is relatively low, and it is unclear whether damage is caused by the direct attack of the virus or by secondary damage due to changes in certain circulating factors caused by COVID-19 damage to other organs. A prior study examined the expression levels of ACE2 protein in human organs and tissues such as the nasopharynx, lung, stomach, and small intestine, and found that ACE2 is most enriched in the small intestine, and lung 8. Although ACE2 receptors have been found in certain cells of the bone marrow, the effects of the virus on the biological activity of bone tissue has not yet been investigated.

MicroRNAs (miRNAs) naturally exist in a variety of living organisms, acting as a crucial element in gene modulation through binding to a specific region in open reading frames (ORFs) or untranslated regions (UTRs), blocking the translation process either by degrading or blocking the mRNA and leading to a knock down or through suppression of downstream processes 9-11. It has been reported that overexpression of miR-29a can result in myocardial injury 12. Furthermore, overexpression of miR-483-3p was demonstrated to contribute to the dysfunction of endothelial progenitor cells 13. Thus, abnormal miRNA expression may be responsible for the COVID-19-related organ damage.

COVID-19 is currently controlled in China and research has begun on assessing the secondary organ damage caused by COVID-19. In our research we aim to explore the biological alteration of bone remodeling in patients who have previously been infected with COVID-19, and to establish whether these patients are more likely to develop osteoporosis later in life.

## Results

### COVID-19 was not detected in the bone tissue of orthopaedic patients

In the post-pandemic era, a large number of orthopaedic patients, including IgG(+) patients, flooded into hospitals, exerting great pressure on the medical system. Prior studies had shown that SARS-CoV-2 could still be detected in patients with IgG(+) who had recovered 14. Since up to 50% of throat swabs for SARS-CoV-2 can be false negatives, further screening for SARS-CoV-2 is of great significance 15. Prior to the admission of orthopaedic patients, nucleic acid testing, pulmonary computer tomography (CT) and temperature assessment were performed. Subsequently, to explore whether there were any viral residues, we recruited 50 IgG (-) and 30 IgG (+) fracture patients, and collected muscle, bone and bone marrow specimens during surgery for nucleic acid testing. Our results showed that no SARS-CoV-2 residues were seen in the muscle, bone or bone marrow specimens of the patients, indicating that orthopaedic surgery on IgG (-) and IgG (+) patients in the post-pandemic era is safe and reliable (Table 1).

### Differentially expressed miRNAs between IgG (-) and IgG (+) patients

**Figure 1:**
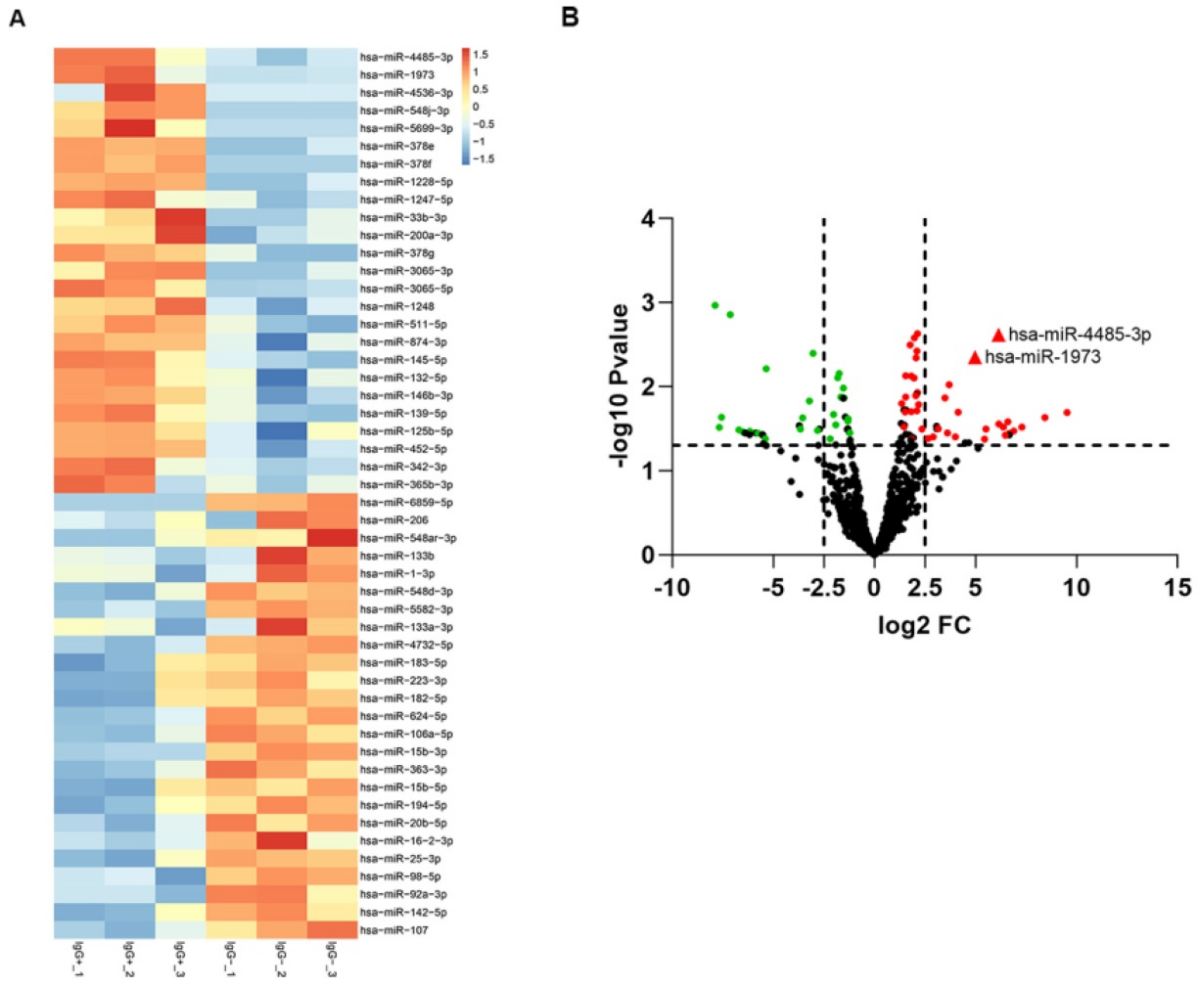
** The differentially expressed miRNAs between IgG(+) and IgG(-) patients.** (A) The cluster analysis diagram and (B) volcano diagram of the differentially expressed miRNAs.

**Figure 2:**
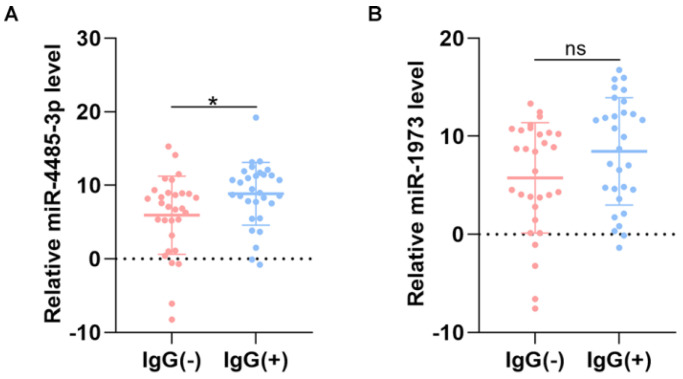
** The level of miRNAs between IgG(+) and IgG(-) patients.** (A) The level of miR-4485-3p between IgG(+) and IgG(-) patients. (B) The level of miR-1973 between IgG(+) and IgG(-) patients. n=30, per group. Data are the mean ± SD 3 independent experiments. *p<0.05, **p<0.01, ***p<0.001.

To screen out any miRNAs differentially expressed (DEM) in IgG (-) patients compared to the IgG (+) patients, a miRNA microarray method was used to compare their miRNA expression profiles. Significantly distinct miRNA profiles were identified between IgG (-) and IgG (+) patients (Fig. 1A-B). Subsequently, a mean fold change>5 or <-5 and a p value <0.01 was set to identify the DEM. According to this criteria, miR-4485-3p and miR-1973 were selected as the top two overexpressed DEMs. The level of the top two miRNAs were further detected via qRT-PCR in thirty independent IgG(-) and IgG(+) patients, and the result suggested that miR-4485-3p was significantly upregulated in IgG(+) patients compared to the IgG(-) patients (Fig. 2A-B).

### Overexpression of miR-4485-3p suppresses osteogenic differentiation

**Figure 3:**
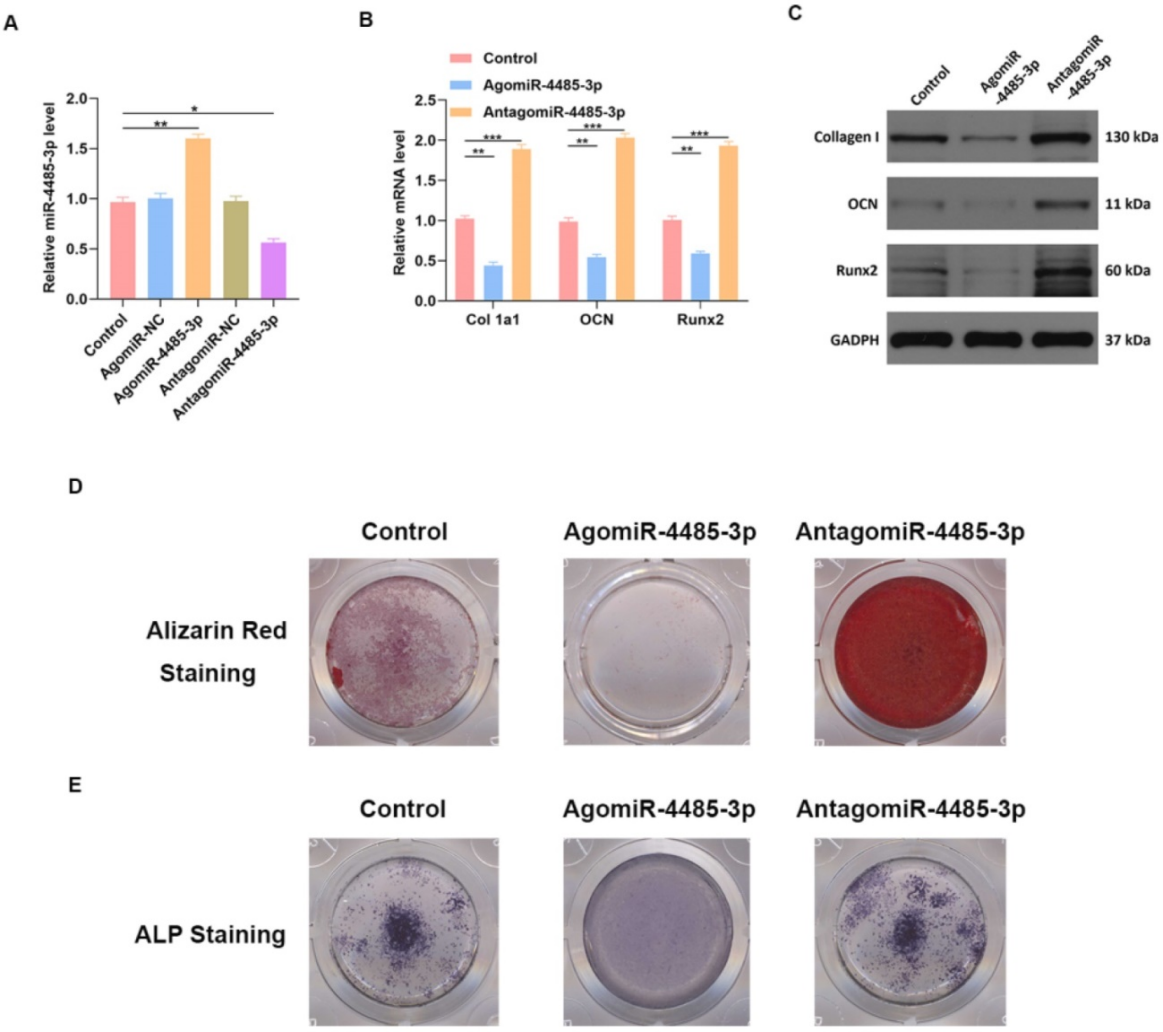
** miR-4485-3p suppress osteogenic differentiation *in vitro*.** (A) The level of miR-4485-3p was measured by qRT-PCR analysis. (B) The level of osteogenic-related mRNAs in different groups was measured by qRT-PCR analysis. (C) The level of osteogenic-related mRNAs in different groups was measured by western blot analysis. (D-E) The alizarin red staining and ALP staining results of different groups. Data are the mean ± SD 3 independent experiments. *p<0.05, **p<0.01, ***p<0.001.

We investigated the ability of miR-4485-3p to directly affect bone marrow mesenchymal stem cells (BMSCs) by treating the cells with phosphate buffer saline (PBS), control agomiR construct (agomiR-NC), agomiR-4485-3p, control antagomiR construct (antagomiR-NC), or antagomiR-4485-3p. Significant elevation of the miR-4485-3p was found in the BMSCs after agomiR-4485-3p treatment (Fig. 3A). Furthermore, the level of osteogenic differentiation-related genes, such as collagen I, osteocalcin (OCN), and Runt-related transcription factor 2 (Runx2), was assessed using western blotting and qRT-PCR analysis, which showed a clear suppression of these genes in the agomiR-4485-3p-treated group (Fig. 3B-C). Moreover, to explore the regulatory role of miR-4485-3p in the extracellular matrix mineralization process, cells were cultured for 21 days, and alizarin red staining was conducted, and the result indicted an enhanced mineral deposition in the agomiR-4485-3p-treated group (Fig. 3D). Similarly, overexpression of miR-4485-3p induced more pronounced ALP activity and staining (Fig. 3E and Fig. S1).

### Toll-like receptor 4 (TLR4) is a potential target gene for miR-4485-3p

**Figure 4:**
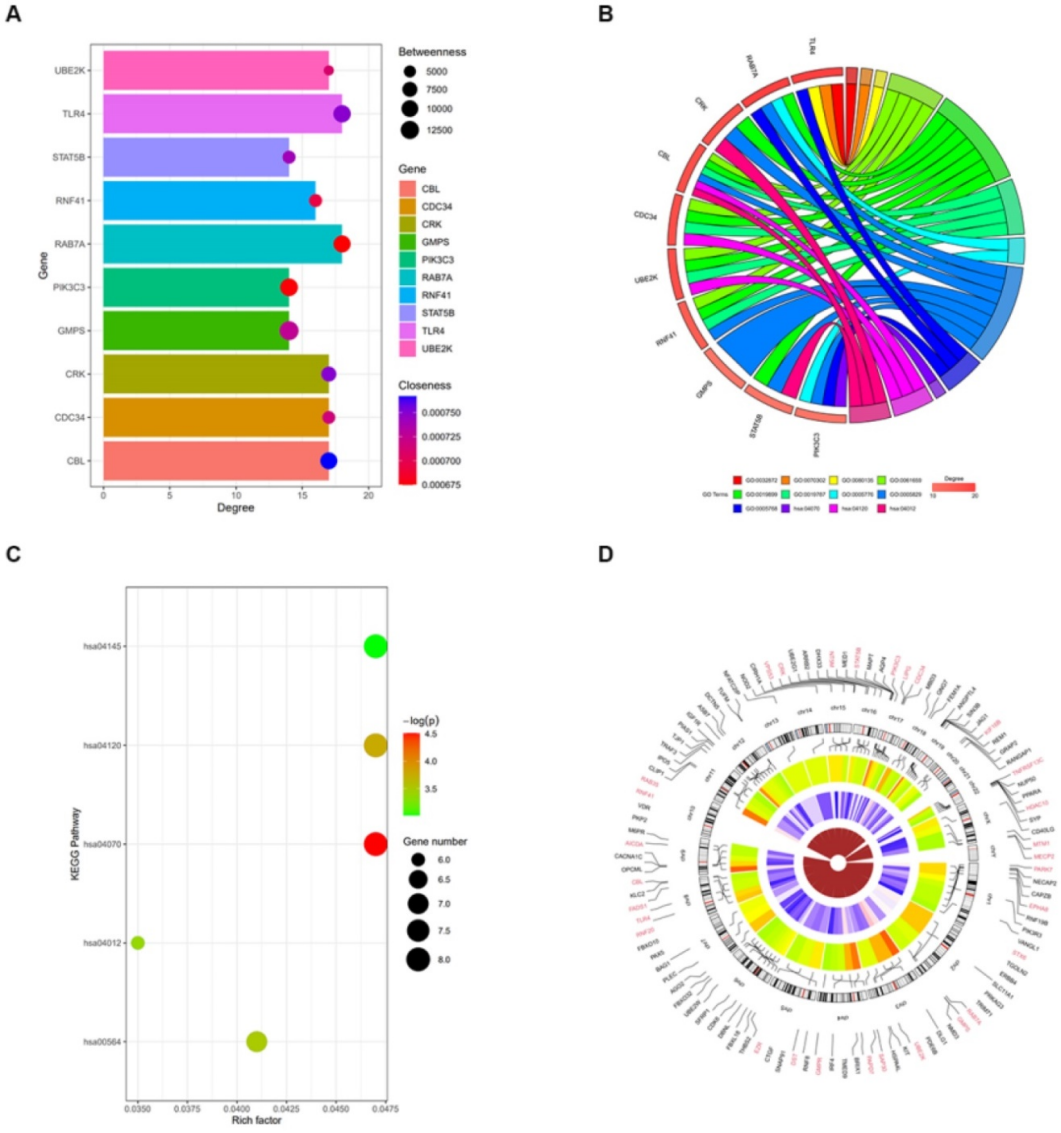
**Toll-like receptor 4 (TLR4) is a potential target gene for miR-4485-3p. (A)** The top 10 hub genes with the highest degree of interaction determined with Cytoscape (Centiscape 2.2 plugin) were CBL, CDC34, CRK, GMPS, PIK3C3, RAB7A, RNF41, STAT5B, TLR4, and UBE2K (Fig. 4A). (B-C) The top 10 genes associated with the enriched GO/KEGG pathway are illustrated using a chord diagram. (D) Expression of the top 50 genes from the 6 modules and their positions on chromosomes are also shown.

We further assessed the molecular mechanisms through which miR-4485-3p inhibits BMSC differentiation. The TargetScan predicting tool was used to identify putative miR-4485-3p targets. The potential target gene list of miR-4485-3p was downloaded and, using STRING, was used to construct a protein-protein-interaction (PPI) network. The top 10 hub genes with the highest degree of interaction determined with Cytoscape (Centiscape 2.2 plugin) were CBL, CDC34, CRK, GMPS, PIK3C3, RAB7A, RNF41, STAT5B, TLR4, and UBE2K (Fig. 4A). The top 10 genes associated with the enriched GO/KEGG pathway are illustrated using a chord diagram (Fig. 4B-C). Expression of the top 50 genes from the 6 modules and their positions on chromosomes are also shown (Fig. 4D).

### TLR4 is involved in miR-4485-3p-mediated osteogenic differentiation

**Figure 5:**
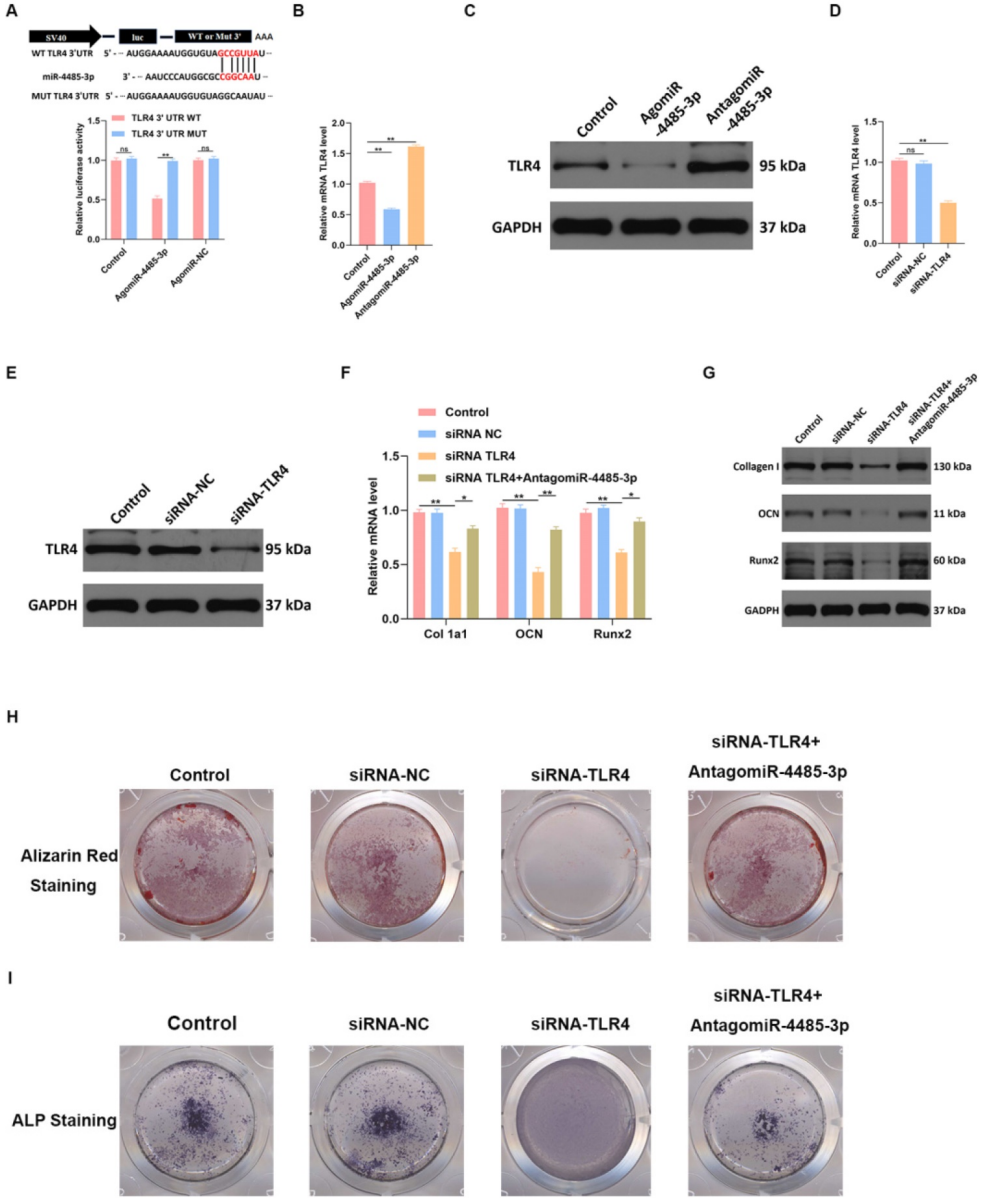
** TLR4 is involved in miR-4485-3p-mediated osteogenic differentiation.** (A) The luciferase assay of miR-4485-3p and TLR4. (B-E) The level of TLR-4 was measured by qRT-PCR and western blot analysis. (F-G) The level of osteogenic-related mRNAs in different groups was measured by western blot analysis. (H-I) The alizarin red staining and ALP staining results of different groups. Data are the mean ± SD 3 independent experiments. *p<0.05, **p<0.01, ***p<0.001.

To detect the binding ability of miR-4485-3p and TLR-4, a luciferase reporter assay was conducted, which showed miR-4485-3p was capable of binding to the predicted target region of the TLR4 mRNA, and the miRNA was no longer able to bind to suppress luciferase activity when these regions were mutated (Fig. 5A). Moreover, a clear inhibition of TLR4 was found in the BMSCs after agomiR-4485-3p treatment (Fig. 5B-C). To investigate the dependence of osteoblastogenesis on the TLR4 gene, we used a TLR4-specific small interference RNA (siRNA) to examine its role in the process (Fig. 5D-E). The qRT-PCR and western blotting results indicated that TLR4 knockdown resulted in elevated collagen I, OCN, and Runx2 expression while miR-4485-3p inhibition could partially restore the expression of the osteogenic-related genes (Fig. 4F-G). Moreover, we further investigate the impact of TLR4 gene on extracellular matrix mineralization. After growth for 21 days in BMSC-inducing conditional media, weaker mineral deposition was seen in the cells treated with TLR4-specific siRNA relative to a control, and the treatment of miR-4485-3p inhibition could partially rescue the adverse effect of decreased TLR4 on osteogenic differentiation (Fig. 5H and Fig. S2). Similarly, TLR4-specific siRNA weakened the ALP activity and staining (Fig. 5I).

### Overexpression of miR-4485-3p hinders fracture healing *in vivo*

**Figure 6:**
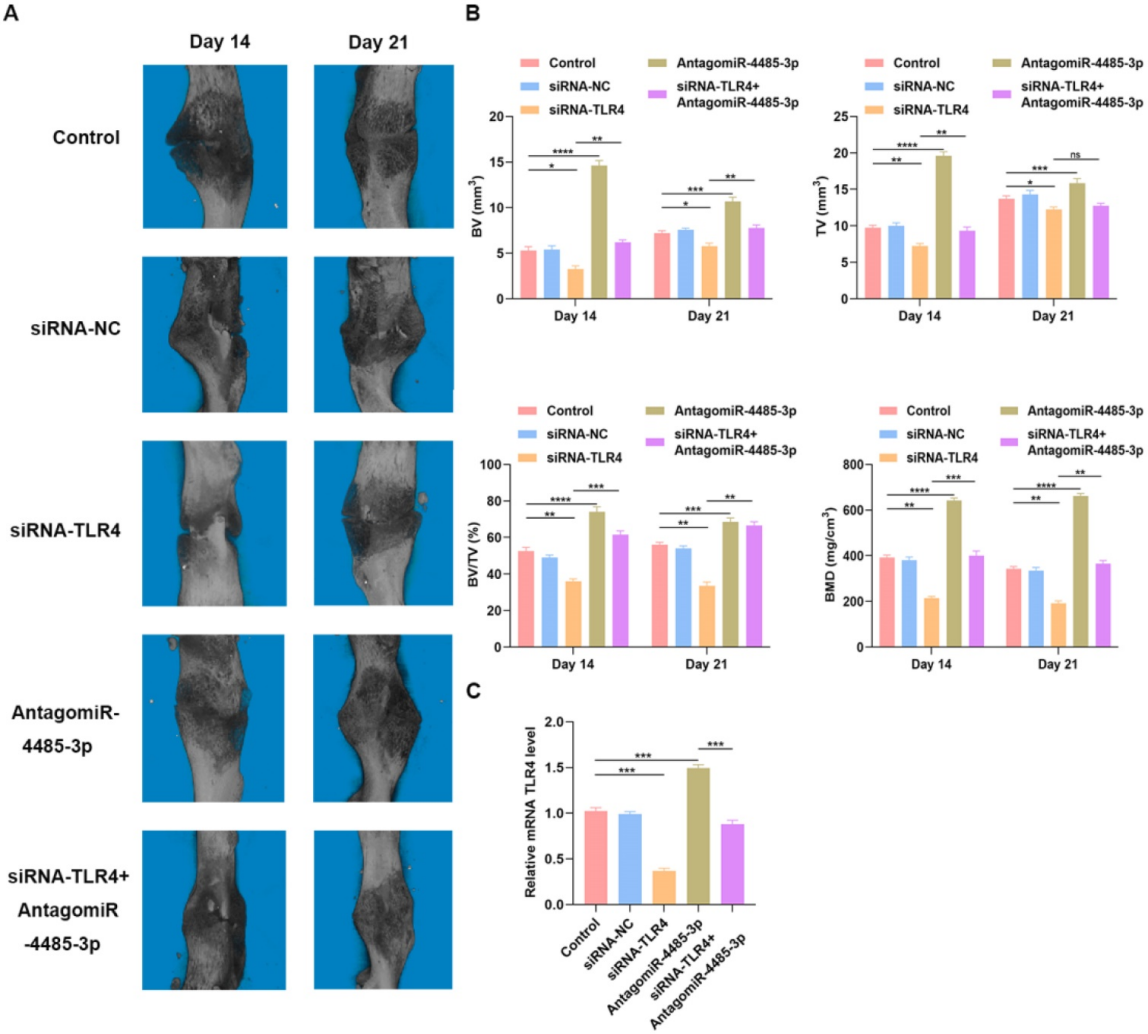
** Overexpression of miR-4485-3p hinders fracture healing *in vivo*.** (A) The animal micro-CT results of different groups. (B) The analysis of micro-CT parameters. (C) The level of TLR4 in the callus was assessed by qRT-PCR analysis, n=6, per group. *p<0.05, **p<0.01, ***p<0.001.

To detect how SARS-CoV-2-induced overexpression of miR-5106 affects fracture healing, we administered PBS, siRNA-NC, siRNA-TLR4, AntagomiR-4485-3p, or siRNA-TLR4+AntagomiR-4485-3p directly to the fracture site in our murine model system on days 1, 4, and 7 post-injury. Animals were monitored using s micro-CT. A significantly reduced fracture gap and higher callus volume was noted in mice treated using AntagomiR-4485-3p, while a significantly larger fracture gap and smaller callus volume was noted in mice treated with siRNA-TLR4, compared to the control group. On day 21 post-fracture, animals treated with AntagomiR-4485-3p no longer exhibited a clear boundary between hardened callus and cortical bone, and remodeling had taken place. When adding siRNA-TLR4, the positive effect was reversed (Fig. 6A-B). Furthermore, on day 14, bone samples were collected for qRT-PCR analysis of the mRNA TLR level, revealing that levels of TLR4 were significantly decreased in the siRNA-TLR4 groups relative to those in the other treatment groups, and AntagomiR-4485-3p treatment could partially restore the TLR4 expression in animals (Fig. 6C).

### Local delivery of miR-4485-3p antagonist rescues the delayed fracture healing phenotype in mice

**Figure 7:**
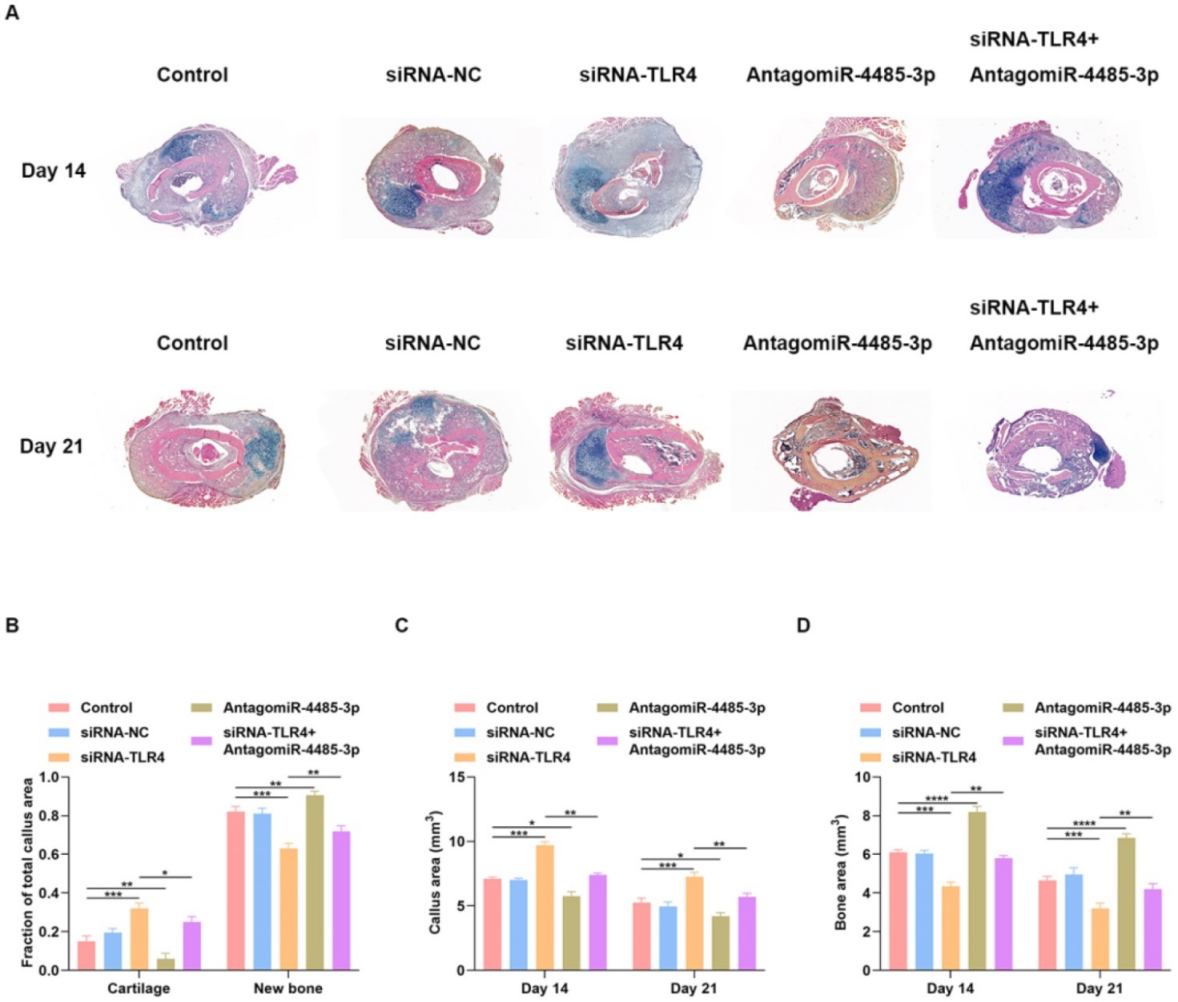
** Local delivery of miR-4485-3p antagonist rescues the delayed fracture healing phenotype in mice. (A)** Fractures in the siRNA-TLR4+AntagomiR-4485-3p-treated mice had reduced cartilage and increased bone compared with siRNA-TLR4-treated control fractures. (B) Histomorphometry showed that fractures in the AntagomiR-4485-3p-treated mice were composed of a markedly high proportion of bone, and low proportion of cartilage. In contrast, fractures in the siRNA-TLR4-treated mice were composed of low bone density and high cartilage density. (C-D) siRNA-TLR4-treated fractures in mice had increased callus area and decreased bone area compared with AntagomiR-4485-3p-treated mice. *p<0.05, **p<0.01, ***p<0.001.

We examined whether local delivery of AntagomiR-4485-3p can compensate for the delayed fracture repair that occurs in siRNA-TLR4-treated mice. PBS, siRNA-NC, siRNA-TLR4, AntagomiR-4485-3p, or siRNA-TLR4+AntagomiR-4485-3p was applied directly to the fracture site in our murine model system on days 1, 4, and 7 post-injury. Tissues harvested after 14 days showed better healing in the AntagomiR-4485-3p-treated mice with minimal remaining cartilage and abundant bone formation (Fig. 7A). Fractures in the siRNA-TLR4+AntagomiR-4485-3p-treated mice had reduced cartilage and increased bone compared with siRNA-TLR4-treated control fractures (Fig. 7A). Histomorphometry showed that fractures in the AntagomiR-4485-3p-treated mice were composed of a markedly high proportion of bone, and low proportion of cartilage. In contrast, fractures in the siRNA-TLR4-treated mice were composed of low bone density and high cartilage density (Fig. 7B). Furthermore, siRNA-TLR4-treated fractures in mice had increased callus area and decreased bone area compared with AntagomiR-4485-3p-treated mice (Fig. 7C-D). Thus, AntagomiR-4485-3p appears to accelerate the endochondral phase of bone repair in mice and results in a relatively rapid fracture healing.

## Discussion

Since the first case of COVID-19 was diagnosed in Wuhan, China, in December 2019, COVID-19 has spread globally becoming a pandemic 17. The majority of COVID-19-related damage occurs in the pulmonary system, manifesting as typical pulmonary infection symptoms, including cough, shortness of breath, fever, and fatigue 18. Furthermore, ACE-2 signaling is widely accepted to contribute to the progression of COVID-19, although the specific mechanism remains elusive 19, 20. Other organs rich with ACE-2 receptors, such as brain, liver, and heart, have also been demonstrated to be main locations of COVID-19 damage 21, 22. A recent report indicated that patients with COVID-19 can also have neurologic injury and manifestations 23. However, research on the damage caused by COVID-19 on organs with low levels of ACE-2 receptors is sparce, and it is unclear whether this damage is caused directly by the virus, or through indirect injury due to alteration of the regulators in circulation.

A recent study investigated the expression of ACE-2 receptors in a wide variety of human tissues, and it was reported that ACE-2 expression is lowest in the blood, spleen, bone marrow, brain, blood vessels, and muscle 24. To the best of our knowledge, there has yet to be any research published on the effect of COVID-19 on the biological activity of bone metabolism. Thus, we aimed to investigate the effects of COVID-19 on bone remodeling and fracture healing, and explore whether COVID-19 can predispose patients infected with the virus to osteoporosis. RNA sequencing was performed on IgG (+) patients who had been infected with SARS-CoV-2 and control IgG (-) patients. miR-4485-3p was identified as the most differentially expressed miRNA which was highly enriched in IgG (+) patients. Prior research reported that miR-4485-3p is involved in the regulation of cell proliferation and apoptosis 25, but few studies have focused on the connection between this miRNA and osteogenic differentiation. In the current study, we demonstrated that miR-4485-5p negatively regulates bone remodeling *in vitro* and *in vivo*. Furthermore, the underlying mechanism of this regulation was also explored, and TLR4 was identified as a potential downstream regulator of miR-4485-3p.

The involvement of TLR4 in the inflammatory response has previously been reported; TLR4 cooperates with LY96 and CD14 to mediate the innate immune response to bacterial lipopolysaccharide (LPS) 26. It acts via MYD88, TIRAP and TRAF6, leading to NF-kappa-B activation, cytokine secretion and an inflammatory response 27. It has been reported that TLR4 is closely linked to osteogenic differentiation of human adipose-derived stem cells through activation of the TLR signaling pathway 28. In the present study, we used the online predicting tool TargetScan to identify putative miR-4485-3p targets. Bioinformatic methods were used to screen out the hub genes among the potential target genes, and TLR4 was one of the top ten hub genes identified as miR-4485-3p targets. Furthermore, the TLR4 reduction induced by the overexpression of miR-4485 was shown to inhibit osteogenic differentiation and bone remodeling. Therefore, TLR signaling appears to be an important molecular mechanism through which miR-4485-3p regulates bone formation.

Taken together, the experiments showed that miR-4485-5p is highly enriched in IgG (+) patients, and, as it is involved in fracture repair, can impact the entire healing cascade. Furthermore, TLR signaling was identified as an important potential therapeutic target for improving fracture healing in the SARS-CoV-2-infected population. In addition, no viral residues were seen in the muscle, bone and bone marrow specimens from the IgG (-) and IgG (+) fracture patients, highlighting that orthopaedic surgery on IgG (-) and IgG (+) patients in the post-pandemic era is safe and reliable.

## Materials and Methods

### Ethical issue

All protocols of the present study were approved by the Ethics Committee of Union Hospital, Tongji Medical College, Huazhong University of Science and Technology (HUST), and informed written consent was obtained from all participants. All experiments involving human samples were conducted in compliance with the Declaration of Helsinki.

### SARS-CoV-2 test

Muscle, bone and bone marrow specimens were collected from 50 IgG (-) and 30 IgG (+) fracture patients. Reverse transcription-PCR (RT-PCR) was performed on the muscle, bone, and bone marrow samples for possible viral shedding routes. The cycle threshold (Ct) value of RT-PCR was used to approximately represented the viral load (inversely related to Ct value) in the patients. Two target genes, open reading frame1ab (ORF1ab) and nucleocapsid (N), were amplified using two sets of primers recommended by the National CDC (China) (https://ivdc.chinacdc.cn/kyjz/202001/t2020 0121_211337.html). IgM and IgG against SARS-CoV-2 were determined using a total blood sample tested with a 2019 nCov IgG/IgM rapid test (Genrui Biotech, Shenzen, China).

### Gene ontology (GO) and Kyoto Encyclopedia of Genes and Genomes (KEGG) pathway analyses

The online tool Database for Annotation, Visualization and Integrated Discovery version (DAVID) Bioinformatics Resources 6.8 was used to perform GO term and KEGG pathway enrichment analysis of the Differentially Expressed Genes (DEGs). The analysis result was visualized with GOplot. The volcano plot was visualized with GraphPrism 8.0.

### BMSCs culture and transfection

Bone marrow stromal cells (BMSCs) were cultured in a specific medium designed for human mesenchymal stem cells (Cyagen, Guangzhou, China) at 37 °C in a 5% CO_2_ incubator. Ipofectamine 2000 (TermoFisher Scientific, USA) was used to transfect cells with siRNAs or miRNAs based on the manufacturer's instructions. AgomiR-/AntagomiR- plasmids (GenePharma, Shanghai, China) were transfected into cells using a 20 μM concentration.

### Luciferase assay

Luciferase plasmid was used to prepare reporter plasmids containing either the wild-type or mutated versions of the 3'-UTR of TLR4. The putative miR-4485-3p binding site was deleted in the mutated version. Cells were co-transfected with these constructs (10 µg) and the appropriate miR-4485-3p agonist using Lipofectamine 2000 according to the manufacture's protocol. Dual-Luciferase Reporter Assay kit was taken to quantify luciferase activity after 48 hours based on the manufacturer's instructions.

### Western blotting (WB) analysis

Samples were diluted at a ratio of 1:5 and heated at 95 °C for 5 minutes. Proteins were separated by SDS-PAGE and transferred to polyvinylidene fluoride membranes. 5% milk was used to block the membranes in TBST for 1 hour at room temperature, incubated with primary antibodies at 4°C overnight, and incubated with horseradish peroxidase-conjugated secondary antibodies at 37 °C for 1 hour. Primary antibodies and dilutions were as follows: anti-TLR4 (1:1000; #ab13556, Abcam, Cambridge, UK), anti-Collagen I (1:1000; #ab138492, Abcam, Cambridge, UK), anti-OCN (1:1000; #ab133612, Abcam, Cambridge, UK), anti-Runx2 (1:1000; #ab236639, Abcam, Cambridge, UK), and anti-GAPDH (1:10000; #ab37168, Abcam, Cambridge, UK). Secondary antibodies were used to probe the blots, and protein was detected with a LiDE110 scanner (Canon, Tokyo, Japan).

### qRT-PCR assessment

A SeraMir Exosome RNA Purification Kit (System Biosciences, Mountain View, CA, USA) was used to extract miRNAs, followed by the use of a TaqMan microRNA assay kit (Applied Biosystems, Foster City, USA) for cDNA synthesis. qRT-PCR was performed using a StepOne Real-Time PCR System (Life Technologies, Carlsbad, CA, USA). Relative gene expression was calculated using the 2^-ΔΔCT^ approach, and GAPDH was used for normalization. The primer sequences used for qRT-PCR are shown in **Table 2**.

### Alizarin red staining

Human bone marrow stromal stem cells were cultured in a special medium containing 100 nM dexamethasone, 50 mM ascorbic acid, and 10 mM B-glycerophosphate (Cyagen, USA). After 2 weeks, cells were washed twice and fixed in 10% formalin for 15 minutes. For staining process, 1 mL 0.5% alizarin red dye solution was added and sustained for 15 minutes at room temperature. The red mineralized nodules were analyzed with a charge coupled device detector, after rinsing with distilled water for 5 minutes. Absorbance was then measured at 570 nm. Experiments were repeated in triplicate.

### ALP staining

BCIP/NBT ALP color staining kit (Beyotime, China) was used based on the provided instructions. Firstly, cells were washed twice with PBS, and then add 10% formalin to fix the cells for 15 minutes. The cells were then treated with BCIP/NBT substrate for 24 hours, the colorimeter changes were analyzed by charge coupled microscopy, and the stained cells were imaged with a scanner. Absorbance was then measured at 405 nm. Experiments were repeated in triplicate.

### Animals and treatments

50 male C57BL/6 mice (8 weeks old, 20-25g body weight) were anesthetized by intraperitoneal injection of sodium pentobarbital (50 mg/kg, Sigma-aldrich, Missouri, USA). As previously reported, we modeled a femoral fracture through a longitudinal incision and blunt dissection of the underlying muscle without removing the periosteum. The femur was incised with a diamond disc to form an intermediate shaft transverse osteotomy. Next, a number 23 intramedullary needle was used to stabilize the fracture site. The mice were randomly divided into five groups that received different treatments. On day 1, 3, and 7 after the fracture, the mice were injected locally at the fracture site (100 μL per site). On day 14 after the fracture, 50% of the study animals were euthanized to harvest and analyze the calluses, and the remaining mice were euthanized and analyzed on day 21.

### Micro‑CT analysis

The fractures in the mice were imaged using a SkyScan 1276 miniature CT system. The platform software was used to evaluate the segmentation, 3D morphological analysis, density and distance parameters. After scanning, the preserved calluses were kept at -80 °C for further experiments. The CT analysis software (version 1.15.4.0) was used to analyze measurement parameters including bone volume (BV), total volume (TV), BV/TV, and bone density (BMD) on the 14^th^ and 21^st^ day after surgery. MicroCT analysis was performed in a completely blinded manner and all mice were assigned to a coded sample number.

### Histology and analysis

Mice were euthanized 14 and 21 days after fracture. A normal middle femur was used as a non-fracture control on day 0. The femur was dislocated from the hip joint and trimmed to remove all excess muscle and skin, which were kept for two days in 10% neutral buffer formalin. The tissue was infiltrated and embedded in paraffin, and Alcian blue, orange G and hematoxylin-Eosin (HE) were used for staining. Histological analysis used standard eyepiece mesh to measure the tissue area within the fracture callus. The total area of the external callus and the area of individual tissue types such as new bone (mineralized tissue) and total cartilage were quantified. The new bone was defined as any new area of woven bone, cartilage tissue was stained blue by proteoglycans, and cortical bone was excluded from histological analysis.

### Statistical analysis

The experiment was independently repeated at least 3 times, with at least 3 samples in each group. Data are expressed as mean ± standard deviation (SD). Student T test was used for comparison between the two groups, and one-way ANOVAs was used for comparison between the two groups and above. All analyses were performed using GraphPad Prism 8.0 (GraphPad Software, San Diego, CA, USA).The significance threshold is P<0.05.

## Supplementary Material

Supplementary figures.

## Figures and Tables

**Table 1:** COVID-19-related results of bone tissues in surgical orthopaedic patients

Patient Number	Age (y)	Gender (M/F)	CT examination (N/A)	Nuclei acids test	IgG	IgM	Bone marrow *ORFlab*	Bone marrow *nCoV-N*	Muscle *ORFlab*	Muscle *nCoV-N*	Bone *ORFlab*	Bone *nCoV-N*
1	61	F	N	(-)	(-)	(-)	(-)	(-)	(-)	(-)	(-)	(-)
2	45	F	N	(-)	(-)	(-)	(-)	(-)	(-)	(-)	(-)	(-)
3	54	M	N	(-)	(-)	(-)	(-)	(-)	(-)	(-)	(-)	(-)
4	77	M	N	(-)	(-)	(-)	(-)	(-)	(-)	(-)	(-)	(-)
5	59	F	N	(-)	(-)	(-)	(-)	(-)	(-)	(-)	(-)	(-)
6	74	F	A	(+)	(++)	(+)	(-)	(-)	(-)	(-)	(-)	(-)
7	53	F	N	(-)	(-)	(-)	(-)	(-)	(-)	(-)	(-)	(-)
8	54	M	N	(-)	(-)	(-)	(-)	(-)	(-)	(-)	(-)	(-)
9	37	F	N	(-)	(-)	(-)	(-)	(-)	(-)	(-)	(-)	(-)
10	50	M	N	(-)	(-)	(-)	(-)	(-)	(-)	(-)	(-)	(-)
11	68	M	A	(-)	(-)	(-)	(-)	(-)	(-)	(-)	(-)	(-)
12	85	M	N	(-)	(-)	(-)	(-)	(-)	(-)	(-)	(-)	(-)
13	84	F	N	(-)	(-)	(-)	(-)	(-)	(-)	(-)	(-)	(-)
14	40	F	N	(-)	(-)	(-)	(-)	(-)	(-)	(-)	(-)	(-)
15	46	F	N	(-)	(-)	(-)	(-)	(-)	(-)	(-)	(-)	(-)
16	66	F	A	(+)	(++)	(-)	(-)	(-)	(-)	(-)	(-)	(-)
17	32	M	N	(-)	(-)	(-)	(-)	(-)	(-)	(-)	(-)	(-)
18	71	M	N	(-)	(-)	(-)	(-)	(-)	(-)	(-)	(-)	(-)
19	81	M	N	(-)	(-)	(-)	(-)	(-)	(-)	(-)	(-)	(-)
20	83	M	N	(-)	(-)	(-)	(-)	(-)	(-)	(-)	(-)	(-)
21	35	M	N	(-)	(-)	(-)	(-)	(-)	(-)	(-)	(-)	(-)
22	63	M	N	(-)	(-)	(-)	(-)	(-)	(-)	(-)	(-)	(-)
23	29	F	N	(-)	(-)	(-)	(-)	(-)	(-)	(-)	(-)	(-)
24	20	F	N	(-)	(-)	(-)	(-)	(-)	(-)	(-)	(-)	(-)
25	60	F	N	(-)	(-)	(-)	(-)	(-)	(-)	(-)	(-)	(-)
26	77	M	N	(-)	(-)	(-)	(-)	(-)	(-)	(-)	(-)	(-)
27	82	F	N	(-)	(-)	(-)	(-)	(-)	(-)	(-)	(-)	(-)
28	56	F	N	(-)	(-)	(-)	(-)	(-)	(-)	(-)	(-)	(-)
29	67	F	N	(-)	(-)	(-)	(-)	(-)	(-)	(-)	(-)	(-)
30	60	F	N	(-)	(-)	(-)	(-)	(-)	(-)	(-)	(-)	(-)
31	63	F	N	(-)	(-)	(-)	(-)	(-)	(-)	(-)	(-)	(-)
32	52	M	N	(-)	(-)	(-)	(-)	(-)	(-)	(-)	(-)	(-)
33	56	M	N	(-)	(-)	(-)	(-)	(-)	(-)	(-)	(-)	(-)
34	55	M	N	(-)	(-)	(-)	(-)	(-)	(-)	(-)	(-)	(-)
35	84	F	N	(-)	(-)	(-)	(-)	(-)	(-)	(-)	(-)	(-)
36	76	M	N	(-)	(-)	(-)	(-)	(-)	(-)	(-)	(-)	(-)
37	76	M	N	(-)	(-)	(-)	(-)	(-)	(-)	(-)	(-)	(-)
38	50	F	N	(-)	(-)	(-)	(-)	(-)	(-)	(-)	(-)	(-)
39	58	F	N	(-)	(-)	(-)	(-)	(-)	(-)	(-)	(-)	(-)
40	35	F	N	(-)	(-)	(-)	(-)	(-)	(-)	(-)	(-)	(-)
41	80	M	N	(-)	(-)	(-)	(-)	(-)	(-)	(-)	(-)	(-)
42	71	M	N	(-)	(-)	(-)	(-)	(-)	(-)	(-)	(-)	(-)
43	53	F	N	(-)	(-)	(-)	(-)	(-)	(-)	(-)	(-)	(-)
44	61	M	N	(-)	(-)	(-)	(-)	(-)	(-)	(-)	(-)	(-)
45	26	F	N	(+)	(++)	(+)	(-)	(-)	(-)	(-)	(-)	(-)
46	27	F	N	(-)	(-)	(-)	(-)	(-)	(-)	(-)	(-)	(-)
47	44	F	N	(-)	(-)	(-)	(-)	(-)	(-)	(-)	(-)	(-)
48	27	F	N	(-)	(-)	(-)	(-)	(-)	(-)	(-)	(-)	(-)
49	64	M	N	(-)	(-)	(-)	(-)	(-)	(-)	(-)	(-)	(-)
50	29	M	N	(-)	(-)	(-)	(-)	(-)	(-)	(-)	(-)	(-)
51	35	M	N	(-)	(-)	(-)	(-)	(-)	(-)	(-)	(-)	(-)
52	27	F	N	(-)	(-)	(-)	(-)	(-)	(-)	(-)	(-)	(-)
53	41	M	N	(-)	(-)	(-)	(-)	(-)	(-)	(-)	(-)	(-)

Abbreviation: y, years; M, Male; F, Female; N, Normal; A, Abnormal.

**Table 2:** microRNAs and mRNA primer sequences

microRNA or Gene Names	Primer sequence (5' to 3')
hsa-miR-4485-3p-Forward	TAACGGTCGCGGTACCCTAA
hsa-miR-4485-3p-Reverse	AGACTGGATGAGACTGTGACTTG
hsa-U6-Forward	CTCGCTTCGGCAGCACA
hsa-U6-Reverse	AACGCTTCACGAATTTGCGT
hsa-TLR4-Forward	TTCGTCATGCTTTCTCACGG
hsa-TLR4-Reverse	ATCAGAGTCCCAGCCAGATG
hsa-Col-1a1-Forward	GAGGGCCAAGACGAAGACATC
hsa-Col-1a1-Reverse	CAGATCACGTCATCGCACAAC
hsa-OCN-Forward	CACTCCTCGCCCTATTGGC
hsa-OCN-Reverse	CCCTCCTGCTTGGACACAAAG
hsa-Runx2-Forward	TGGTTACTGTCATGGCGGGTA
hsa-Runx2-Reverse	TCTCAGATCGTTGAACCTTGCTA
hsa-GAPDH-Forward	GGAGCGAGATCCCTCCAAAAT
hsa-GAPDH-Reverse	GGCTGTTGTCATACTTCTCATGG
