# Supplementary material for: SARS-CoV-2-induced Overexpression of miR-4485 Suppresses Osteogenic Differentiation and Impairs Fracture Healing
Source: Int J Biol Sci. 2021 Mar 25;17(5):1277–88. doi: 10.7150/ijbs.56657 (PMC8040480; doi:10.7150/ijbs.56657)
Supplement: Supplementary file 1 — Supplementary figures. [file ijbsv17p1277s1.pdf]

## Supplementary Materials

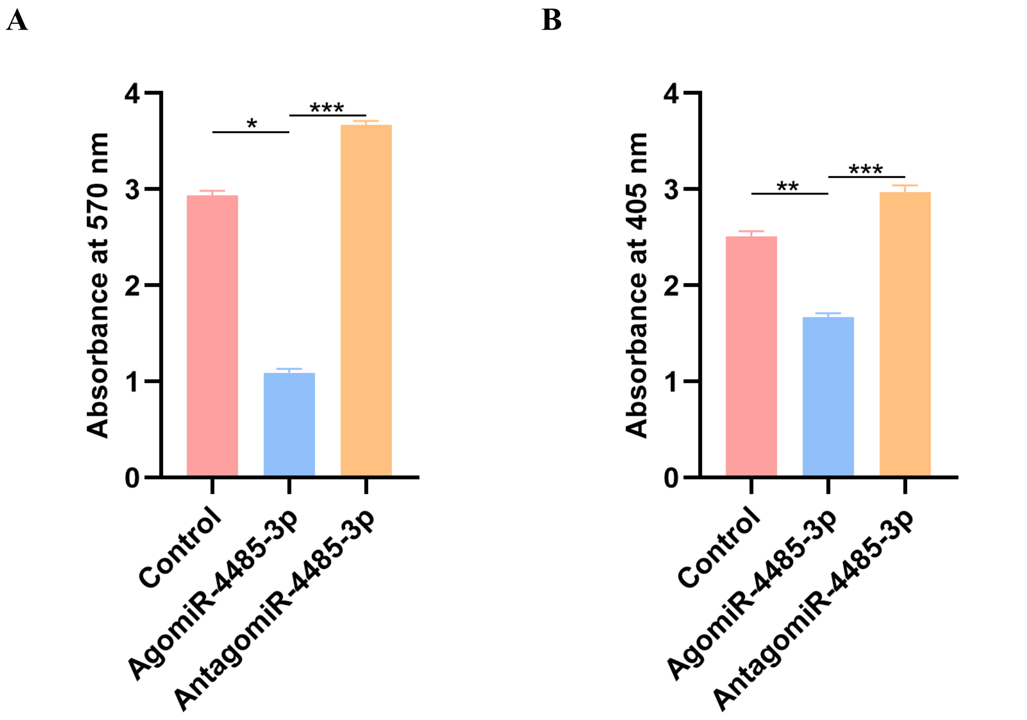


**Figure S1. The quantification of alizarin red-mediated calcium staining (A) and ALP staining (B) in BMSCs following differently transfected. Data are mean±SD. *p<0.05, **p<0.01, ***p<0.001**

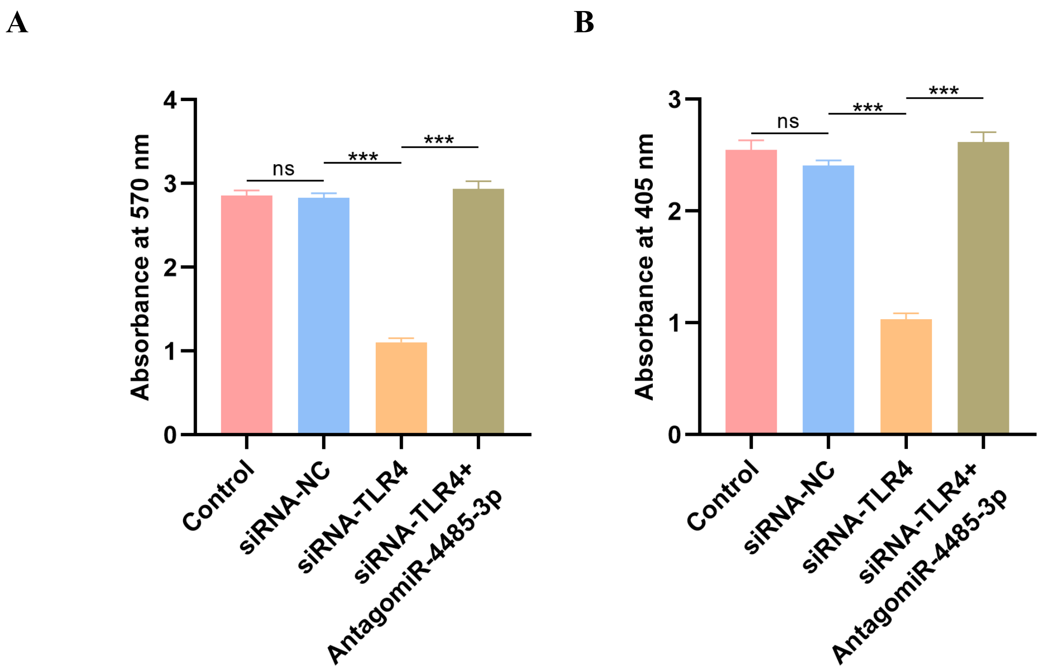


**Figure S2. The quantification of alizarin red-mediated calcium staining (A) and ALP staining (B) in BMSCs following differently transfected. Data are mean±SD. *p<0.05, **p<0.01, ***p<0.001**
